# Nutritional and physicochemical quality of formulations based on colostrum and bovine whey

**DOI:** 10.1371/journal.pone.0267409

**Published:** 2022-05-02

**Authors:** Alyne B. S. Galdino, Katya Anaya, Idiana M. Barbosa, Luís H. F. Borba, Emerson G. S. O. Silva, Cláudia S. Macêdo, Cláudio V. D. M. Ribeiro, Juliana P. F. Oliveira, Adriano H. N. Rangel

**Affiliations:** 1 Postgraduate Program in Animal Production, Federal University of Rio Grande do Norte, Macaíba, RN, Brazil; 2 Faculty of Health Sciences of Trairi, Federal University of Rio Grande do Norte, Santa Cruz, Brazil; 3 Academic Unit Specialized in Agricultural Sciences, Federal University of Rio Grande do Norte, Macaíba, Rio Grande do Norte, Brazil; 4 Animal Science Department, Federal University of Bahia, Salvador, Bahia, Brazil; 5 Rural Health and Technology Center, Federal University of Campina Grande, Patos, Brazil; Universidade Federal de Minas Gerais, BRAZIL

## Abstract

The objective of this study was to investigate the nutritional quality of bovine colostrum and whey mixtures. Five whey with bovine colostrum formulations were prepared (90:10; 80:20; 70:30; 60:40 and 50:50 whey:colostrum v:v) to be subjected to low-temperature pasteurization (63°C to 65°C for 30 minutes) and freeze-drying. The samples underwent chemical composition characterization, fatty acid profile analysis, determination of contamination by Enterobacteriaceae, pH, and Dornic acidity measurements before and after vat pasteurization. The amount of protein, fat, total solids, defatted dry extract, Brix and density increased as the bovine colostrum concentration increased. The level of saturated fatty acids and the thrombogenicity and atherogenicity indices reduced, while unsaturated fatty acids increased as the level of added bovine colostrum increased. The low-temperature pasteurization of the formulations was possible and effective, eliminating contamination by Enterobacteriaceae in the samples. Mixing bovine colostrum and whey reduced the colostrum viscosity, allowing a successful pasteurization procedure. Due to colostrum composition, the formulations yielded a higher nutritional value when compared to whey alone. The parameters applied in the formulation of mixtures of bovine colostrum and whey resulted in valuable ingredients for preparing novel dairy products.

## Introduction

In bovine, colostrum can be secreted for up to 3–5 days, depending on a variety of internal conditions [1], and its composition differs from whole milk. Lactose is decreased in colostrum, and tends to increase over the days until it reaches mature milk levels. The amount of protein, as well as casein, immunoglobulins, albumin and lactoferrin, are higher in colostrum compared to milk [2]. The same happens with lipids, in which there is a reduction in their levels up to 14 days postpartum [3]. On average, more than 70% of the fatty acids in bovine colostrum are saturated, while 20% are monounsaturated and 5% polyunsaturated [4]. Growth factors, mainly IGF-I, IGF-II and TGF-β1, immune modulators IL-1β, IL-6, TNF-α, INF-γ, and immunoglobulins IgA, IgG and IgM are also at higher levels in colostrum. Regarding minerals and vitamins, colostrum has an increased content of calcium and fat-soluble vitamins [1, 2, 5]. These components make colostrum a potentially beneficial bioactive food for human health [6].

In turn, whey is a co-product resulting from the coagulation of casein, mainly for cheese production [7]. Its volume as a surplus is considered high, because 9 L of whey is produced for every 1 kg of cheese [8]. However, it is made up of 6.5% of total solids, of which 0.8% is protein (20% of all milk protein), 0.5% fat and 4.5% lactose, in addition to minerals such as calcium [9, 10]. The main proteins present are β-lactoglobulin, α-lactalbumin, casein, immunoglobulins, lipoprotein, bovine serum albumin (BSA), lactoferrin, lactoperoxidase, bioactive peptides and essential amino acids [9, 11]. It also contains fractions of cell growth factors, such as IGF-I, IGF-II, TGF-β1 and TGF-β2 [12]. These compounds provide whey with important bioactive properties for human health, such as immunomodulatory, antimicrobial, and prebiotic functions. These effects are possible because these compounds remain active after passing through the gastrointestinal tract, and may exert their functions in the large intestine [13].

However, bovine colostrum and whey are still undervalued in milk-producing properties and in dairy industries, respectively. This is because although they are rich in nutrients, a part of its surplus is usually discarded [14–16]. Whey was initially largely devalued and considered a waste by the dairy industry. It only gained commercial value and began to be better reused after the prohibition of its untreated disposal and recognition of its nutritional value and the functionality of its constituents [17], although it is not completely reused due to its high production volume [14].

Although the composition of colostrum has aroused the interest of industries for manufacturing innovative functional foods [1], several issues limit its use in some countries. The wide compositional variety, uncertain availability, lack of adequate preservation technologies, ethical and regulatory issues are some of the reasons that hinder its use by industries [15]. Furthermore, its low coagulation temperature combined with the fragility of its constituents to heating hinder essential processes such as pasteurization [18].

Until the time that this study was carried out, there were no previous reports of other studies that investigated the possibility of industrial bovine colostrum use associated with whey. Thus, in aiming to increase the use of its nutrients, the objective of this work was to study the physicochemical and nutritional composition, as well as the fatty acid composition and the microbiological quality of formulations based on colostrum and bovine whey before and after pasteurization in order to investigate the stability of the mixture to pasteurization.

## Material and methods

### Obtaining and collecting bovine colostrum and bovine whey

Colostrum was collected from Jersey cows at the third milking, between 24 and 48 hours post-calving, in a commercial farm located in São Gonçalo do Amarante, Rio Grande do Norte, Brazil, between July and November 2020. After being collected, the colostrum was stored under freezing conditions at -20°C and transported in coolers to physicochemical composition and colostrum quality analyses and processing to prepare the formulations carried out.

The bovine whey was originated from *coalho* cheese production with whole milk, which took place at the Escola Agrícola de Jundiaí, of the Federal University of Rio Grande do Norte (*UFRN*). After coagulating the milk with a rennet enzyme and separating the cheese mass, the whey was heated at 65°C for 30 minutes, filtered and stored under freezing at -20°C for further analysis of physicochemical composition and processing to prepare the formulations.

### Developed formulations

Five formulations of the bovine colostrum with liquid bovine whey mixture were tested in the proportions 90:10, 80:20, 70:30, 60:40 and 50:50 (whey:colostrum v:v), then named F10, F20, F30, F40 and F50, respectively.

### Procedures for processing and freeze-drying the bovine colostrum + whey mixtures

Bovine colostrum and whey were initially thawed under refrigeration for 24 hours. After thawing, a sample of each was taken for analysis, while the rest was used in preparing the formulations. To do so, colostrum and whey were mixed following the proportions mentioned above. The mixtures were homogenized and an aliquot of the material was removed for analysis before the formulations were taken to pasteurization.

Low-temperature pasteurization was performed in a water-bath, with parameters from 63°C to 65°C for 30 minutes, as stated in Decree No. 9013, of March 29, 2017 [19]. Temperatures above that could coagulate colostrum or even cause greater losses of its bioactive constituents [20]. The mixtures were immediately cooled after 30 minutes, and kept refrigerated until performing the analysis, which took place within 24 hours after pasteurization.

For the freeze-drying process, 100 mL of homogenized pasteurized and unpasteurized liquid samples were frozen on plates at a temperature of -20°C for 72 hours and then placed in a freeze-dryer (Liobras, model L101, São Paulo, Brazil). The drying process took place for 48 hours, at a pressure of less than 500 μHg, and the result was a yellowish coloured powder. After being removed from the apparatus, the material was placed under vacuum and stored away from light for further analysis.

**Fig 1:**
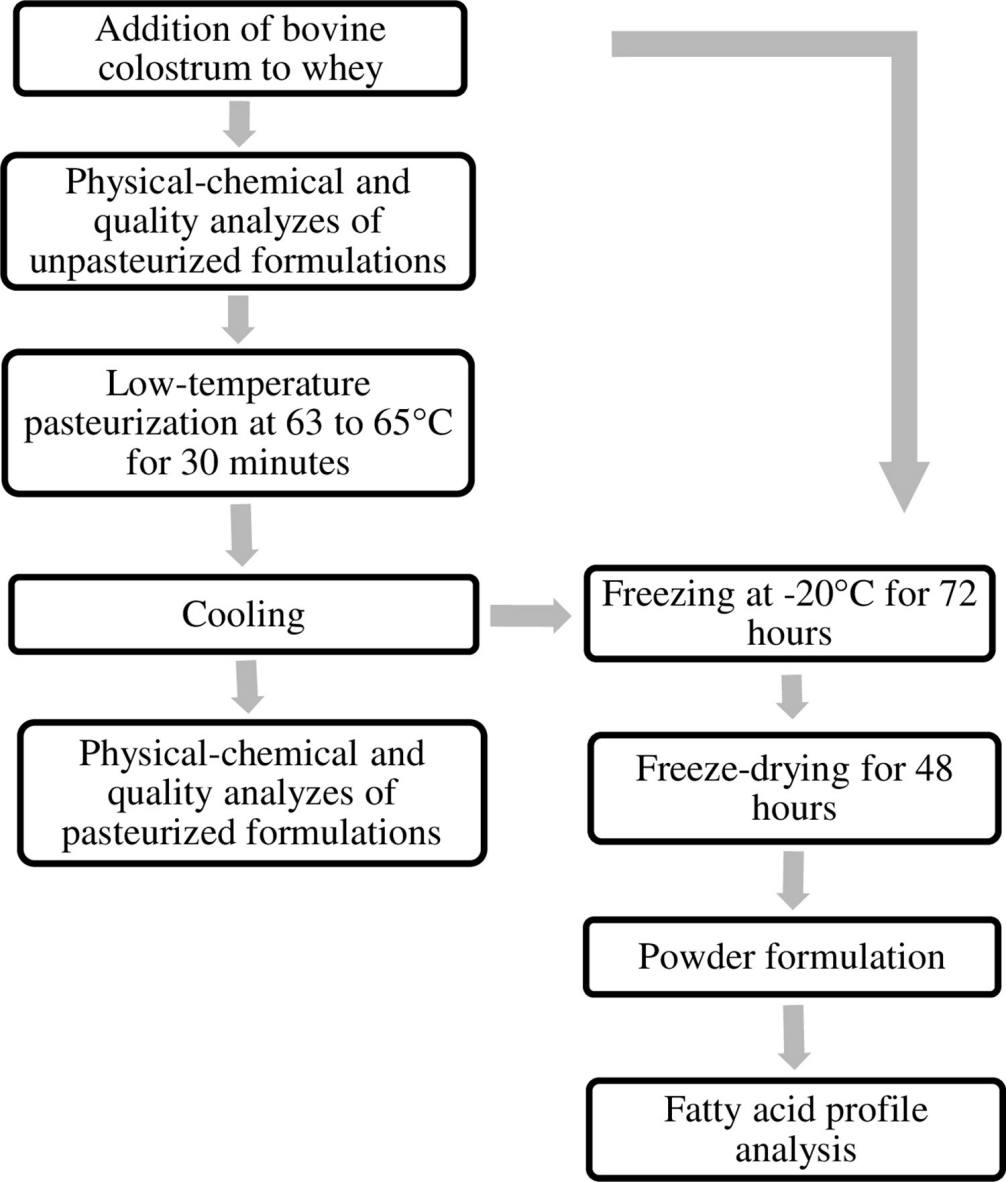
Processing flowchart for the production and pasteurization of formulations and freeze-drying of pasteurized and unpasteurized samples for fatty acid profile analysis.

A flowchart of the formulations’ production is illustrated in Fig 1, from adding the colostrum to the whey to producing the mixture of freeze-dried powder to analyze the fatty acid profile.

### Analysis of the chemical composition of samples

The physicochemical composition of colostrum, whey and mixtures was determined before and after pasteurization. Protein, fat, total solids and defatted dry extract were determined by infrared absorption method (Dairy Spect^®^, Bentley Instruments Inc., Chaska, Minnesota, USA).

### Physicochemical analysis of pH and Dornic acidity

The pH of colostrum, whey and mixtures was measured using a digital pH meter (Lucadema, model LUCA-210, São Paulo, Brazil) calibrated according to the manufacturer’s recommendations.

Acidity was determined by titration with Dornic solution. To do so, 10 mL of the samples were placed in different Erlenmeyer flasks. Then, 5 drops of phenolphthalein were added and homogenized. Finally, the samples were titrated with Dornic solution until their turning point [21]. The results were expressed in g of lactic acid/100 mL.

### Analysis of the specific gravity of colostrum and formulations using the colostrometer

A colostrometer (Kruuse^®^, Denmark) was used to analyze the quality of the colostrum and tested mixtures. Thus, 250 mL of colostrum and mixtures were placed in a beaker and the colostrometer was inserted, allowing it to float freely. The reading was performed after stabilizing the device in the sample, recording the scale immediately above the submerged part. Samples with a density on the colostrometer above 1.046 are considered to be of excellent quality, from 1.036 to 1.046 of moderate quality, and below 1.036 of inferior quality [22].

### Brix analysis

A drop of colostrum and the mixtures was placed using sterile Pasteur pipettes in the prism of an optical refractometer (Kasvi, model-k52-032, China) for the refractometer analysis. The reading result was expressed in %Brix.

### Density analysis

The density of the mixtures was analyzed using a densimeter. To do so, 250 mL of the formulations was placed in a beaker and subsequently inserted into the densimeter which floated freely in the samples. After the device was stabilized, the scale was recorded immediately above the submerged part. The verified value was applied in a conversion table to determine the density of the samples.

### Microbiological analysis

Due to the lack of specific legislation for pasteurized bovine colostrum, the limit recommended by Normative Instruction No. 76 of November 26, 2018, which determines a maximum limit of 5 CFU/mL of Enterobacteriaceae for pasteurized milk, was followed. Therefore, microbiological analyzes of the Enterobacteriaceae family (CFU/mL) were performed in bovine colostrum whey and formulations before and after pasteurization to determine the effectiveness of pasteurization. Thus, Compact Dry ETB chromogenic plates (HyServe^©^, Germany) were used, in which 1 mL of the diluted sample was applied to the center of the plate. The plates were subsequently closed and incubated at 37°C for 24 hours. The colonies were then counted after the incubation time according to the manufacturer’s recommendations.

### Fatty acid analysis

The lipid profile of the freeze-dried samples was determined at the Multifunctional Laboratory of the School of Veterinary Medicine and Animal Science of the Federal University of Bahia, using the methodology adapted from [23]. Approximately 0.5g of the samples were placed in glass tubes with screw caps internally lined with Teflon, to which 2 mL of hexane and 2 mL of sodium methoxide (0.5M in methanol) were added, with subsequent vortexing for 30 seconds and heating in a water bath at 50°C for 10 minutes. Afterwards, 3 mL of acetyl chloride (5% in methanol) were added to be heated again to 80°C for 10 minutes. The tubes were then cooled and 1 mL of hexane and 10 mL of 6% K_2_CO_3_ were added, vortexing them for 1 minute and centrifuging them for 2 minutes. The supernatant was transferred to tubes containing 1.0 g of Na_2_SO_4_ (oven dried), which were shaken for 30 seconds and centrifuged for another 1 minute. The supernatant was collected and transferred to chromatography tubes and subsequently stored in a freezer at -20°C.

The quantification of fatty acid methyl esters was performed in a gas chromatograph (Focus GC—Thermo Scientific) equipped with a flame ionization detector (CG-DIC) and SPTM—2560 capillary column (100 mx 0.25 mm x 0.20 μm—Supelco). The initial oven temperature was programmed to 140°C, increasing at a heating rate of 1°C/min up to 220°C, remaining at this temperature for 25 minutes [24]. The carrier gas was hydrogen gas at a flow of 1.5 mL/minute. Next, 1 μL of the extract was injected into the device. Injections occurred in duplicate for each extract. The retention times of the sample peaks were then compared with the retention time of the reference standard esters (GLC-674, Nu-Chek Prep, Inc.) to identify the fatty acid methyl esters, and the results were expressed as a percentage.

The following formulas were used to determine the thrombogenicity (TI) and atherogenicity (AI) indices [25]:

$$TI=\frac{\left(C14:0+C16:0+C18:0\right)}{\left[(0.5*AGMI)+(0.5*n6)+(3*n3)+(n3/n6)\right]}$$ (1)

$$AI=\frac{\left[C12:0+(4*C14:0)+C16:0\right]}{\left(AGMI+n6+n3\right)}$$ (2)

### Statistical analysis

Analysis of variance was used to test the effects of the formulations on the parameters evaluated. When significant, the comparison of means between formulations was performed using the Tukey’s test (p < 0.05). Dunnett’s test (p < 0.05) was used to compare means of whey and bovine colostrum with the tested formulations. The SAS version 9.0 software program was used for the statistical analyzes.

## Results and discussion

### Physicochemical and quality characteristics

There was an enrichment of protein, fat, defatted dry extract (DDE) and consequently of total solids as the bovine colostrum percentage increased (Table 1). According to Normative Instruction No. 76, of November 26, 2018, raw and pasteurized milk must have at least 2.9 g/100g of protein in its composition [26]. Only the F10 formulation (90:10, whey:colostrum) did not meet this parameter, presenting from 2.71 to 2.79 g/100g of protein. Thus, four of the five tested formulations have adequate protein content for use in producing dairy products which usually use milk for their preparation. However, the F10 formulation could still be used for manufacturing derivatives that require low protein content, as in the case of dairy drinks. Normative Instruction No. 16, of August 23, 2005, defines that dairy beverages must have at least 1.0 to 1.7g/100g of protein, depending on the type of dairy beverage produced [27].

**Table 1:** Chemical composition (g/100g) of liquid formulations according to the addition of bovine colostrum and pasteurization, and comparison of the chemical composition of the formulations with the chemical compositions of bovine colostrum and whey.

	Composition (g/100g)			
	Fat	Protein	Total solids	DDE
**Bovine colostrum**	3.38±1.02	4.30±0.55	16.08±1.00	11.75±1.47
**Whey**	0.46±0.04	2.39±0.09	7.49±0.28	6.50±0.23
**Non-pasteurized**				
**F10**	0.36±0.08^c^*	2.71±0.07^c^*	8.32±0.11^e.B^**	7.36±0.17^c^*
**F20**	0.75±0.19^b.c^*	2.91±0.10^b.c^**	9.32±0.12^d^**	7.92±0.27^c^**
**F30**	1.12±0.28^a.b.c^**	3.11±0.14^b.c^**	10.28±0.14^c^**	8.47±0.38^b.c^**
**F40**	1.43±0.34^a.b^**	3.32±0.18^a.b^**	11.23±0.21^b^**	9.07±0.50^a.b^**
**F50**	1.76±0.43^a^**	3.55±0.23^a^**	12.24±0.25^a^**	9.69±0.62^a^**
**Pasteurized**				
**F10**	0.34±0.10^c^*	2.79±0.04^d^**	8.53±0.06^e.A^**	7.57±0.11^d^**
**F20**	0.71±0.18^b.c^*	2.97±0.10^c.d^**	9.45±0.11^d^**	8.09±0.27^c.d^**
**F30**	1.07±0.26^a.b.c^*	3.18±0.13^b.c^**	10.43±0.13^c^**	8.66±0.36^b.c^**
**F40**	1.41±0.35^a.b^**	3.38±0.16^a.b^**	11.36±0.14^b^**	9.20±0.43^a.b^**
**F50**	1.72±0.42^a^**	3.58±0.22^a^**	12.33±0.16^a^**	9.82±0.51^a^**

DDE: Defatted dry extract. Means followed by different lowercase letters in the same column within the same heat treatment differ from each other by Tukey’s test (p < 0.05). Means followed by different capital letters in the same column differ by Tukey’s test (p < 0.05).

*samples differ from bovine colostrum by Dunnett’s test (p < 0.05)

**samples differ from bovine colostrum and whey by Dunnett’s test (p < 0.05).

Milk proteins are important for producing dairy products. They are responsible for the structure, appearance, texture, viscosity and mouthfeel of these foods [28]. The applicability of its functional properties, such as foaming, aeration, viscosity formation, gelling, emulsification, solubility, among others, varies according to the type of dairy product you want to produce [28, 29]. However, these properties depend on the structural characteristics of proteins, which can be affected by extrinsic and environmental factors, such as pH and temperature [30]. The union of colostrum proteins with those of whey may have caused important changes in the protein profile of these substances, as well as modified their levels compared to colostrum and whey.

All formulations had reduced fat content when compared to whole milk (minimum of 3.0 g/100g), with the F10 formulation being closer to skimmed milk, which can have a maximum of 0.5 g/100g of fat, and the others closer to semi-skimmed milk which can have from 0.6 to 2.9 g/100g of fat [26]. As whey has low fat content, its association with bovine colostrum was effective in reducing the fat content of the latter without the need to use expensive technologies for defatting. The protein remained high and close to the protein content found in whole milk (3.0 g/100g) [26], ranging from 2.71 to 3.58 g/100ml between pasteurized and unpasteurized formulations (Table 1).

The total solids of the formulations were statistically different from each other (p < 0.05). This indicates that each formulation can also have a different industrial application. According to Normative Instruction No. 76, of November 26, 2018, whole milk must contain a minimum content of 8.4g/100g of non-fat solids and at least 11.4g/100g of total solids [26]. Among the developed formulations, only F30, F40 and F50 presented non-fat solids contents above the minimum for whole milk, while only F40 and F50 presented total solids contents close to or above 11.4 g/100 g. Derivatives such as dairy drinks and ricotta cheese, for example, come from a mixture of milk with whey, and therefore require less total solids for their manufacture [31, 32]. On the other hand, hard cheeses, curds, firmer yoghurts and several other derivatives need milk with higher solids content, so that they obtain better yield and greater firmness [33, 34]. Therefore, when testing such formulations in the development of new products, it is important to pay attention to their proximate composition and the characteristics of the expected product, and not only to the higher nutritional value. In this context, the F10 and F20 formulations are more suitable for producing derivatives which require lower levels of total solids, while the F30, F40 and F50 could be used in manufacturing those that need higher levels of total solids.

Regarding the pasteurization effects, only the F10 formulation (90:10 whey:colostrum v:v) showed statistical difference in its total solids content between pasteurized and unpasteurized. Protein denaturation may occur during whey heating due to their molecules unfolding, or even the formation of protein complexes resulting from other existing protein fractions aggregation [35]. These changes can cause changes in the structure of important bioactive components of whey [36]. [37] observed smaller reductions of lactoferrin and transforming growth factor β (TGF-β) in sera that underwent milder pasteurization.

Heating also causes bovine colostrum protein denaturation and consequently the loss or reduction of its bioactive activity [15]. [38] found a reduction in the concentrations of lactoferrin and IGF-1 after heating bovine colostrum at 60°C for 60 minutes and 63°C for 30 minutes, and immunoglobulins at 63°C for 30 minutes.

Furthermore, studies have shown that heating bovine colostrum to temperatures above 60°C can increase its viscosity, resulting in a consistency close to that of pudding [39, 40]. Pasteurization is one of the most used and effective strategies to ensure the microbiological quality of milk used in producing dairy products [41], and therefore this colostrum characteristic has hindered its processing for use in industry.

In this study, low-temperature pasteurization was carried out in accordance with Brazilian legislation for bovine milk with temperatures between 63 and 65°C for 30 minutes [19], and no change in consistency was observed. This demonstrates that diluting bovine colostrum in a compatible substance, such as whey, can facilitate its industrial processing. However, as heat treatment can change the structure of proteins, denaturing or aggregating them, reducing bioactive compounds from both colostrum and whey, it is important that these alterations be investigated in formulations by further studies.

There was no statistical difference between the pH and quality measured with a colostrometer between the different formulation levels, pasteurized or not, nor in the acidity of the unpasteurized formulations, although there were among the pasteurized formulations. There was a gradual increase in Brix and density according to the increase in bovine colostrum in the formulation in both heat treatments (Table 2).

**Table 2:** Physicochemical and quality characteristics of the formulations according to the addition of bovine colostrum and comparison with the physicochemical characteristics of bovine colostrum and whey.

	pH	Acidity (g of lactic acid/100 mL)	Brix (%)	Specific gravity (mg/mL)	Density (g/mL)
**Bovine colostrum**	6.20±0.19	0.40±0.04	17.16±2.47	1.048±0.005	-
**Whey**	6.65±0.28	0.14±0.01	-	-	-
**Non-pasteurized**					
**F10**	6.59±0.24	0.19±0.05*	8.00±0.00^c^*	1.029±0.002*	1.028±0.001^c^
**F20**	6.69±0.48	0.21±0.04*	9.16±0.28^b.c^*	1.033±0.005*	1.029±0.001^b.c^
**F30**	6.78±0.34	0.24±0.03**	10.33±0.58^a.b^*	1.033±0.002*	1.031±0.002^a.b.c^
**F40**	6.67±0.40	0.26±0.04**	11.17±0.76^a^*	1.036±0.001*	1.034±0.003^a.b^
**F50**	6.54±0.17	0.29±0.04**	12.16±1.15^a^*	1.037±0.003*	1.036±0.003^a^
**Pasteurized**					
**F10**	6.57±0.25	0.16±0.01^d^*	8.16±0.29^c^*	1.031±0.003*	1.029±0.001^c^
**F20**	6.32±0.06	0.20±0.02^c.d^**	9.33±0.58^b.c^*	1.033±0.004*	1.030±0.001^b.c^
**F30**	6.27±0.11	0.24±0.01^b.c^**	10.33±0.58^a.b.c^*	1.034±0.002*	1.032±0.002^a.b.c^
**F40**	6.23±0.09	0.26±0.03^a.b^**	11.50±1.32^a.b^*	1.036±0.002*	1.034±0.002^a.b^
**F50**	6.42±0.23	0.30±0.02^a^**	12.50±1.32^a^*	1.037±0.003*	1.036±0.003^a^

Means with different letters in the same column within the same heat treatment differ from each other by the Tukey’s test (p < 0.05).

*samples differ from bovine colostrum by Dunnett’s test (p < 0.05)

**samples differ from bovine colostrum and whey by Dunnett’s test (p < 0.05).

The mean pH of bovine colostrum in our study was within the expected range (6.20±0.19), since according to the literature it can range from 6.00 to 6.61 [2, 42] (Table 2). The whey had an average pH of 6.65±0.28 (Table 2), which is consistent with sweet whey made from enzymes [43].

On the other hand, pasteurized F20 formulations (80:20 whey:colostrum v:v) had higher acidity than pasteurized milk, which can be from 0.14 to 0.18 g of lactic acid/100 mL [26]. This is explained by the fact that colostrum can present a titratable acidity of 2 to 2.5 times greater than that of whole milk [44], and this increase may be associated with its higher protein content [2, 45]. In our study, the mean titratable acidity of colostrum was 0.40±0.04 g of lactic acid/100 ml (Table 2). The acidity of the whey was also within the expected range for sweet whey, being 0.14±0.01 g of lactic acid/100 mL (Table 2). According to [46], the acidity in this type of whey can vary from 0.08 to 0.14 g of lactic acid/100 mL. Thus, since the whey acidity is lower than that of colostrum, it is expected that there will also be an increase in acidity in the formulations with an increase in the colostrum concentration.

The colostrometer and the Brix refractometer are two indirect methods used to measure the quality of bovine colostrum in terms of immunoglobulin (Ig) levels [47]. Colostrum measured by the colostrometer is considered of high quality when it presents specific gravity greater than 1.046 (>50 mg/mL of Ig), of moderate quality when in the range of 1.036 to 1.046 (20–50 mg/mL of Ig), and of low quality when less than 1.036 (< 20 mg/ml Ig) [48]. The disadvantage of this method is that the colostrum temperature can affect the equipment reading, which must always be within the temperature range specified by the manufacturer [47]. The reading performed by the refractometer is independent of colostrum temperature, and therefore becomes more accurate to determine immunoglobulin levels. Good quality colostrum is generally considered when Brix is greater than 21% (> 50 mg/mL of IgG) [49]. However, [50] recommended a specific cut-off point of 18% for Jersey cow colostrum.

In our study, the mean specific gravity of colostrum measured with a colostrometer was 1.048±0.005 (Table 2), characterizing it as high quality (> 50mg/mL of Ig). However, the mean brix degree of 17.16±2.47 (Table 2) was below the 18% determined by [50] for the Jersey breed.

We noticed that there was a progressive increase in the brix degree with the increase in the colostrum percentage regarding the formulations (p < 0.05). This effect is not perceived by the specific gravity measured with a colostrometer, since there was no statistical difference between the formulations (p > 0.05) (Table 2). However, the result expressed by the refractometer can be considered more reliable than that indicated by the colostrometer. This is why [50] found that freeze-thaw cycles could affect the accuracy of specific gravity measured by the colostrometer, an effect which was not seen in the Brix refractometer. The colostrum underwent a freeze-thaw cycle in our study, constituting a factor which may have reduced the colostrometer’s sensitivity in measuring the formulations.

Density measured with a densimeter is one of the main quality parameters of bovine milk, which has an average density of 1.032 [51]. According to Brazilian legislation, raw milk must contain a relative density between 1.028 and 1.034 at 15°C [26]. The density in the formulations was also progressive with the addition of bovine colostrum and all, with the exception of F50 (50:50 whey: colostrum v:v), presented density within what is established in Brazil for milk (Table 2). In turn, F50 had a higher density than milk, which characterizes it with a richer nutritional composition of total solids. This characteristic indicates that this formulation could be tested in manufacturing foods that often require enriching milk in total solids.

By comparing the formulations with the bovine colostrum composition, we can observe that all formulations obtained statistically different content from the colostrum. Regarding whey, we noticed that the F10 formulation had similar protein and fat (p > 0.05), but different total solids (p < 0.05). On the other hand, F20 and F30 only presented protein similar to that of whey (p < 0.05), while F40 and F50 presented different nutritional composition from that of whey (p < 0.05). Importantly, total solids for all formulations were different from colostrum and whey (p < 0.05) (Table 1). These data show that the union of whey and colostrum nutritionally favors whey, while reducing colostrum viscosity, which can facilitate its use in food production.

There was no statistical difference in the pH of the mixtures with bovine colostrum and whey (p > 0.05). There was a progressive increase in acidity as the whey colostrum concentration increased. However, even the formulation with the highest acidity (F50) showed a statistical difference with the acidity of colostrum (p < 0.05). Thus, it is suggested that the combination of bovine colostrum and whey can be efficient in reducing colostrum acidity. In turn, the F10 and F20 formulations showed similar acidity to whey (p > 0.05) (Table 2). Finally, all formulations showed reduced quality compared to colostrum due to the decrease in the Brix degree and specific gravity measured with a colostrometer.

However, it is noteworthy that due to the rich composition of bovine colostrum and whey in bioactive nutrients, it is suggestive that these formulations still have important nutritional content for human health, regardless of the reduction in quality compared to colostrum. Its contents of lactoferrin, TGF-β, immunoglobulins, α-lactalbumin, bioactive peptides, essential amino acids, growth factors, vitamins, and minerals (among others) should be investigated [2, 13, 52, 53].

It is also noteworthy that the effluents from the dairy industries are highly polluting for the environment when untreated due to their high Biological Oxygen Demand (BOD) and Chemical Oxygen Demand (COD) [54]. Therefore, seeking alternatives for the use of excess colostrum and whey volumes is also important from an environmental point of view. For example, whey production has increased in recent years, which has increasingly stimulated the search for its use in order to minimize the environmental contamination caused by its disposal [52]. In addition, the high nutritional quality of colostrum and whey has encouraged their use as raw materials in manufacturing beverages and functional foods for human consumption [6, 11].

### Fatty acid analysis

Tables 3 and 4 show the fatty acid profile and fatty acid groups of pasteurized and unpasteurized formulations, respectively. Table 5 shows the fatty acid profile of pasteurized and unpasteurized bovine colostrum.

**Table 3:** Fatty acid profile of pasteurized and unpasteurized formulations.

Item	Treatments^1^										SEM^2^	P-Value^3^	
	Raw (g/100g)					Pasteurized (g/100g)						Past^4^	Inclusion
	10	20	30	40	50	10	20	30	40	50			
**C14:0**	10.32^d^	9.90^c^	9.60^b^	9.28^a^	9.17^a^	10.35^d^	9.76^c^	9.49^b^	9.05^a^	9.12^a^	0.058	0.481	< 0.001
**C14:1**	0.71^c^	0.65^b^	0.62^b^	0.58^a^	0.57^a^	0.72^c^	0.64^b^	0.62^b^	0.57^a^	0.57^a^	0.008	0.423	< 0.001
**C16:0**	30.95^a^	31.18^ab^	31.38^b^	31.56^b^	31.78^b^	30.53^a^	31.18^ab^	31.58^b^	31.83^b^	31.65^b^	0.129	0.737	< 0.001
**C16:1**	1.82^a^	1.98^b^	2.12^c^	2.16^cd^	2.21^d^	1.78^a^	1.98^b^	2.13^c^	2.17^cd^	2.19^d^	0.017	0.300	< 0.001
**C18:0**	11.63^c^	10.96^b^	10.34^ab^	10.31^ab^	10.18^a^	11.54^c^	10.89^b^	10.54^ab^	10.56^ab^	10.33^a^	0.118	0.430	< 0.001
**C18:1t11**	1.27^d^	1.22^cd^	1.15^bc^	1.03^ab^	0.94^a^	1.33^d^	1.26^cd^	1.13^bc^	1.10^ab^	0.99^a^	0.030	0.985	< 0.001
**C18:1c9**	21.81^a^	23.44^b^	24.72^c^	25.47^cd^	25.98^d^	21.50^a^	23.67^b^	24.87^c^	25.93^cd^	25.96^d^	0.214	0.922	< 0.001
**C18:1c11**	0.63^a^	0.75^b^	0.84^c^	0.86^d^	0.90^d^	0.63^a^	0.77^b^	0.83^c^	0.90^d^	0.90^d^	0.011	0.106	< 0.001
**C18:2n6**	1.89	1.81	1.96	1.97	1.91	1.88	1.95	1.86	1.90	1.94	0.036	0.165	0.187
**C18:3n3**	0.34	0.31	0.29	0.38	0.31	0.59	0.40	0.28	0.32	0.34	0.072	0.184	0.203
**CLAc9t11**	0.35^c^	0.32^b^	0.31^ab^	0.30^a^	0.29^a^	0.34^c^	0.32^b^	0.31^ab^	0.30^a^	0.30^a^	0.004	1.000	< 0.001
**C20:4n6**	0.29^a^	0.37^b^	0.40^c^	0.46^d^	0.48^d^	0.27^a^	0.36^b^	0.42^c^	0.46^d^	0.47^d^	0.008	0.667	< 0.001
**n3**	0.34	0.31	0.29	0.38	0.31	0.59	0.40	0.28	0.32	0.34	0.072	0.184	0.203
**n6**	2.17^a^	2.18^ab^	2.35^ab^	2.42^b^	2.39^b^	2.14^a^	2.31^ab^	2.28^ab^	2.36^b^	2.41^b^	0.037	0.359	< 0.001

^1^Inclusion percentage (10, 20, 30, 40 and 50) of bovine colostrum in whey in raw and pasteurized form.

^2^Standard error of the mean.

^3^Probability of pasteurization effects and colostrum inclusion level on the percentage of fatty acids.

^4^Past: Pasteurized. Means with different letters in the same line within the same heat treatment differ from each other by the Tukey’s test (p < 0.05).

**Table 4:** Fatty acid groups in pasteurized and unpasteurized formulations.

Item	Treatments^1^										SEM^2^	P-Value^3^	
	Raw (g/100g)					Pasteurized (g/100g)						Past	Inclusion
	10	20	30	40	50	10	20	30	40	50			
**SCFA**	4.94^c^	4.76^bc^	4.41^ab^	4.16^a^	3.95^a^	5.33^c^	4.65^bc^	4.29^ab^	3.69^a^	3.96^c^	0.116	0.954	< 0.001
**MCFA**	15.81^d^	14.68^c^	13.87^b^	13.15^a^	12.78^a^	16.18^d^	14.45^c^	13.61^b^	12.68^a^	12.74^a^	0.113	0.462	< 0.001
**SFA**	63.32^d^	61.57^c^	59.99^b^	59.17^a^	58.69^a^	63.58^d^	61.16^c^	60.02^b^	58.75^a^	58.67^a^	0.212	0.893	< 0.001
**UFA**	36.68^a^	38.43^b^	40.01^c^	40.83^d^	41.32^d^	36.43^a^	38.84^b^	39.99^c^	41.25^d^	41.33^d^	0.212	0.893	< 0.001
**n6:n3**	6.55	7.21	8.25	6.52	7.87	4.84	6.02	8.13	7.63	7.39	0.694	0.389	0.069
**TI**	3.67^c^	3.43^bc^	3.23^ab^	3.08^ab^	3.08^a^	3.49^c^	3.31^bc^	3.25^ab^	3.10^ab^	3.06^a^	0.065	0.915	< 0.001
**AI**	2.80^d^	2.57^c^	2.40^b^	2.29^a^	2.24^a^	2.81^d^	2.51^c^	2.38^b^	2.24^a^	2.23^a^	0.026	0.885	< 0.001

^1^Inclusion percentage (10, 20, 30, 40 and 50) of bovine colostrum in whey in raw and pasteurized form.

^2^Standard error of the mean.

^3^Probability of pasteurization effects and colostrum inclusion level on the percentage of fatty acids. Past: Pasteurized; SCFA: Short Chain Fatty Acids; MCFA: Medium Chain Fatty Acids; SFA: Saturated fatty acids; UFA: Unsaturated fatty acids; TI: Thrombogenicity Index; AI: Atherogenicity Index. Means with different letters in the same line within the same heat treatment differ from each other by the Tukey’s test (p < 0.05).

**Table 5:** Fatty acids profile of raw and pasteurized colostrum.

Fatty acid	Raw colostrum (g/100g)	Pasteurized colostrum (g/100g)
**C14:0**	8.62±0.11	8.70±0.05
**C14:1**	0.52±0.01	0.52±0.00
**C16:0**	31.78±0.28	31.89±0.26
**C16:1**	2.34±0.01	2.37±0.02
**C18:0**	9.69±0.11	9.57±0.13
**C18:1t11**	1.06±0.21	1.06±0.25
**C18:1c9**	27.67±0.73	27.60±0.52
**C18:1c11**	0.98±0.01	1.00±0.01
**C18:2n6**	2.00±0.05	2.07±0.00
**C18:3n3**	0.28±0.00	0.30±0.01
**CLAc9t11**	0.27±0.01	0.27±0.01
**C20:4n6**	0.57±0.03	0.56±0.00
**SCFA**	3.60±0.31	3.59±0.11
**MCFA**	11.53±0.21	11.67±0.08
**n3**	0.28±0.00	0.30±0.01
**n6**	2.57±0.08	2.63±0.00
**SFA**	56.59±0.91	56.71±0.57
**UFA**	43.41±0.91	43.30±0.57
**n6:n3**	9.16±0.28	8.78±0.41
**TI**	2.86±0.09	2.85±0.06
**AI**	2.04±0.07	2.05±0.05

SCFA: Short Chain Fatty Acids; MCFA: Medium Chain Fatty Acids; SFA: Saturated fatty acids; UFA: Unsaturated fatty acids; TI: Thrombogenicity Index; AI: Atherogenicity Index.

There was no effect of pasteurization on the fatty acid profile of the formulations. However, the inclusion of bovine colostrum caused different fatty acid profiles among the samples. The saturated fatty acids (SFAs) percentage reduced and unsaturated fatty acids (UFAs) increased as the added bovine colostrum level increased. Omega 6 levels also increased with the addition of bovine colostrum, while omega 3 levels did not differ. The value of TI and AI also reduced as the colostrum concentration was increased. However, bovine colostrum still had lower SFAs and higher UFAs levels, as well as lower TI and AI.

Bovine colostrum SFAs levels were lower than those found by [4] (71.71 g/100 g) and by [55] (more than 60 g/100 g). However, as in the study by [4], the highest SFA percentage in both the formulations and in bovine colostrum was palmitic acid (C16:0).

UFAs represented around 43 g/100 g in bovine colostrum, which is a higher value than that found by [4] (28.29 g/100 g) and by [55] (less than 40 g/100 g). Of these, about 27 g/100 g was oleic acid (C18:0c9), also constituting a higher value than that found by [4] (17.38 g/100 g). The UFAs level in the formulations ranged from 36 to 41 g/100 g, depending on the formulation, also driven by omega 9 (21 to 26 g/100 g).

Omega 6 and omega 3 are essential fatty acids that must be part of the diet, since the human body is not able to synthesize them [56]. However, it is important that there is a balance in the intake of both, as the high consumption of omega 6 associated with low consumption of omega 3 may be related to the development of several pathologies [56–58]. The association of bovine colostrum with whey resulted in a lower n6:n3 ratio than that found in pure bovine colostrum, a factor considered beneficial to human health [57].

The Thrombogenicity Index (TI) assesses the potential of fatty acids in forming clots in blood vessels by the ratio between prothrombogenic fatty acids (C12:0, C14:0 and C16:0) and antithrombogenic acids, monounsaturated fatty acids (MFA) and omega 3 and 6 [59]. Thus, the lower the TI of the food, the greater the benefit to human health [59]. In this study, the TIs of the formulations ranged from 3.06 to 3.67; while it was 2.86 in raw bovine colostrum and 2.85 in pasteurized colostrum. The TI value in the formulations reduced as the colostrum concentration was increased. These values are within those shown by [59] for bovine milk (2.05 to 4.65), and lower than that found for the milk of Jersey cows, which ranged from 4.51 to 4.65 (Salles et al., 2019). The TI value for colostrum was lower than that found by [4] (3.19) and by [60] (4.01).

The Atherogenicity Index (AI) indicates the relationship between the main pro-atherogenic SFAs (C12:0, C14:0 and C16:0), and the UFAs, considered anti-atherogenic because they inhibit plaque accumulation and reduce the phospholipid, cholesterol and esterified fatty acid levels [59]. Thus, the consumption of foods with lower AI values can be beneficial to human health [59]. The formulations in this study presented AI between 2.81 and 2.23, which decreased as the percentage of added bovine colostrum was increased. The AI of raw bovine colostrum was 2.04 and 2.05 for pasteurized colostrum, a value lower than that found by [4] (3.76) and by [60] (3.70). An AI of 4.94 was reported in the literature for milk from Jersey, Dutch and Dutch x Jersey cows at the beginning of lactation (from 3 to 100 days of lactation) [61], and for milk from Jersey cows from 3.18 to 3.43 [62]. Thus, both colostrum and formulations had a lower AI than that reported for milk of the same breed. The values are also within those shown by [59] for bovine milk (1.37 to 5.13).

Thus, pure bovine colostrum had a better fatty acid profile for human health when compared to formulations. However, the formulations still had thrombogenicity and atherogenicity rates close to those found in bovine milk, and the higher the added bovine colostrum level, the lower the rates, so that the F50 formulation demonstrated a better lipid profile for human health.

### Microbiological analysis

*Enterobacteriaceae* is a family of Gram-negative, facultatively anaerobic non-spore-forming rods capable of causing enteric disease in humans. *Salmonella* spp., *Escherichia Coli* and *Yersinia enterocolitica* are some of the species in this family [63].

Pasteurization proved to be efficient for all added bovine colostrum levels. Raw bovine colostrum was contaminated by *Enterobacteriaceae* (1.15 x 10^3^ CFU/mL). It was not possible to identify the *Enterobacteriaceae* species present due to the analysis method adopted. All raw formulations also showed contamination, which was not observed in the pasteurized samples (Table 6). Thus, pasteurization at 63 to 65°C for 30 minutes was efficient in eliminating contamination by *Enterobacteriaceae* in the formulations.

**Table 6:** Microbiological analysis of *Enterobacteriaceae* before and after pasteurization.

Samples	*Enterobacteriaceae* (UFC/ml)	
	Before pasteurization	After pasteurization
**Colostrum**	1.15 x 10^3^	-
**F10**	1.30 x 10^2^	<10.0 est
**F20**	1.40 x 10^2^	<10.0 est
**F30**	2.70 x 10^2^	<10.0 est
**F40**	4.00 x 10^2^	<10.0 est
**F50**	5.90 x 10^2^	<10.0 est

est: estimated.

Colostrum pasteurization at 60°C for 30 minutes and at 55°C for 60 minutes tested by [64] and at 63°C for 30 minutes tested by [65] were not efficient in totally eliminating *Escherichia Coli* contamination in bovine colostrum. However, pasteurization at 60°C for 30 and 45 minutes was efficient in eliminating *Salmonella* spp. from colostrum [20, 66].

## Conclusion

The inclusion of bovine colostrum in whey resulted in mixtures with high amounts of proteins, relatively low fat, and a favorable fatty acid profile, considering the decrease of the thrombogenicity and atherogenicity indices with the increase of bovine colostrum addition. The parameters used in the pasteurization procedure was considered effective, since no technological defects were observed, and the process achieved the desirable *Enterobacteriaceae* elimination. The mixture reduces the bovine colostrum viscosity, which favors its thermal processing and application as a food ingredient for several dairy products. The technological processing of bovine colostrum and whey mixtures was shown as feasible, and the food industry could use it to contribute to a more sustainable food production.

Future studies should focus on investigating the effect of pasteurization on the stability of bioactive molecules in the formulations of whey and bovine colostrum. Further clinical trials would be critical to elucidate the potential beneficial effects of the mixtures, which could suggest their potential to produce functional dairy products.

## Supporting information

S1 Table Data that originated Table 1. (PDF)

S2 Table Data that originated Table 2. (PDF)

S3 Table Data that originated Table 3. (PDF)

S4 Table Data that originated Table 4. (PDF)

S5 Table Data that originated Table 5. (PDF)
