# Supplementary material for: Nutritional and physicochemical quality of formulations based on colostrum and bovine whey
Source: PLoS One. 2022 May 2;17(5):e0267409. doi: 10.1371/journal.pone.0267409 (PMC9060355; doi:10.1371/journal.pone.0267409)
Supplement: S1 Table — (PDF) [file pone.0267409.s001.pdf]

	Repetition	Fat	Protein	Total solids	DDE
Whey	1	0.50	2.49	7.81	6.76
Whey	2	0.42	2.36	7.35	6.41
Whey	3	0.46	2.33	7.30	6.33
Colostrum	1	4.35	4.11	16.50	11.24
Colostrum	2	2.31	4.92	16.81	13.41
Colostrum	3	3.48	3.88	14.94	10.60
F10A	1	0.45	2.63	8.20	7.17
F20A	1	0.94	2.80	9.18	7.62
F30A	1	1.40	2.96	10.12	8.07
F40A	1	1.77	3.13	10.99	8.53
F50A	1	2.21	3.30	11.95	9.01
F10A	2	0.36	2.75	8.38	7.41
F20A	2	0.76	2.94	9.41	8.00
F30A	2	1.14	3.13	10.34	8.51
F40A	2	1.43	3.36	11.33	9.16
F50A	2	1.72	3.61	12.37	9.85
F10A	3	0.29	2.76	8.40	7.50
F20A	3	0.56	2.99	9.36	8.14
F30A	3	0.84	3.24	10.39	8.83
F40A	3	1.09	3.49	11.38	9.52
F50A	3	1.36	3.75	12.41	10.22
F10P	1	0.46	2.74	8.51	7.45
F20P	1	0.90	2.86	9.32	7.79
F30P	1	1.33	3.04	10.28	8.28
F40P	1	1.71	3.22	11.20	8.78
F50P	1	2.14	3.34	12.15	9.26
F10P	2	0.26	2.81	8.49	7.61
F20P	2	0.68	3.00	9.51	8.17
F30P	2	1.09	3.20	10.50	8.70
F40P	2	1.48	3.37	11.42	9.19
F50P	2	1.72	3.64	12.44	9.92
F10P	3	0.32	2.82	8.60	7.66
F20P	3	0.54	3.06	9.52	8.31
F30P	3	0.80	3.30	10.52	8.99
F40P	3	1.03	3.54	11.45	9.64
F50P	3	1.30	3.77	12.40	10.27
