# Supplementary material for: Nutritional and physicochemical quality of formulations based on colostrum and bovine whey
Source: PLoS One. 2022 May 2;17(5):e0267409. doi: 10.1371/journal.pone.0267409 (PMC9060355; doi:10.1371/journal.pone.0267409)
Supplement: S2 Table — (PDF) [file pone.0267409.s002.pdf]

	Repetition	pH	Acidity (g of lactic acid/100 mL)	Brix (%)	Specific gravity (mg/mL)	Density
Whey	1	6.55	14	-	-	-
Whey	2	6.97	15	-	-	-
Whey	3	6.44	12	-	-	-
Colostrum	1	6.35	46	20.0	1.053	-
Colostrum	2	5.98	37	15.5	1.045	-
Colostrum	3	6.26	39	16.0	1.045	-
F10A	1	6.46	25	8.0	1.033	1.0290
F20A	1	6.39	25	9.5	1.038	1.0300
F30A	1	6.40	26	11.0	1.036	1.0332
F40A	1	6.51	27	12.0	1.037	1.0370
F50A	1	6.57	31	13.5	1.041	1.0392
F10A	2	6.87	16	8.0	1.028	1.0280
F20A	2	7.25	17	9.0	1.030	1.0290
F30A	2	7.05	26	10.0	1.033	1.0300
F40A	2	7.13	30	10.5	1.035	1.0320
F50A	2	6.70	24	11.5	1.035	1.0340
F10A	3	6.44	17	8.0	1.028	1.0280
F20A	3	6.45	20	9.0	1.030	1.0290
F30A	3	6.91	20	10.0	1.032	1.0310
F40A	3	6.39	22	11.0	1.035	1.0332
F50A	3	6.36	32	11.5	1.036	1.0350
F10P	1	6.44	18	8.5	1.035	1.0290
F20P	1	6.36	22	10.0	1.038	1.0324
F30P	1	6.30	25	11.0	1.036	1.0344
F40P	1	6.24	29	13.0	1.039	1.0364
F50P	1	6.27	32	14.0	1.041	1.0396
F10P	2	6.85	15	8.0	1.029	1.0290
F20P	2	6.25	18	9.0	1.030	1.0300
F30P	2	6.14	23	10.0	1.032	1.0310
F40P	2	6.14	25	10.5	1.035	1.0330
F50P	2	6.69	28	11.5	1.035	1.0340
F10P	3	6.41	16	8.0	1.029	1.0280
F20P	3	6.35	19	9.0	1.031	1.0300
F30P	3	6.36	24	10.0	1.033	1.0320
F40P	3	6.32	24	11.0	1.035	1.0332
F50P	3	6.31	30	12.0	1.036	1.0352
