# Supplementary material for: Nutritional and physicochemical quality of formulations based on colostrum and bovine whey
Source: PLoS One. 2022 May 2;17(5):e0267409. doi: 10.1371/journal.pone.0267409 (PMC9060355; doi:10.1371/journal.pone.0267409)
Supplement: S3 Table — (PDF) [file pone.0267409.s003.pdf]

	Repetition	C14:0	C14:1	C16:0	C16:1	C18:0	C18:1 t11	C18:1 c9	C18:1 c11	C18:2n6	C18:3n3	CLA c9t11	C20:4n6
F1OA	1	10.35	0.70	31.10	1.82	11.74	1.31	21.48	0.61	1.87	0.30	0.36	0.29
F1OP	1	10.22	0.71	30.44	1.75	11.65	1.28	21.26	0.61	2.00	0.90	0.35	0.28
F2OA	1	9.90	0.64	31.46	1.96	11.20	1.24	23.15	0.73	1.77	0.27	0.33	0.36
F2OP	1	9.74	0.62	31.33	1.97	11.06	1.20	23.34	0.75	1.95	0.32	0.32	0.37
F3OA	1	9.60	0.61	31.68	2.08	10.62	1.15	24.31	0.82	1.95	0.29	0.31	0.42
F3OP	1	9.50	0.61	31.73	2.10	10.67	1.17	24.57	0.83	1.82	0.27	0.31	0.43
F4OA	1	9.27	0.57	31.62	2.12	10.43	0.98	25.10	0.86	1.99	0.44	0.30	0.46
F4OP	1	8.88	0.54	32.09	2.16	10.77	1.13	26.01	0.92	1.93	0.36	0.29	0.48
F5OA	1	9.22	0.56	31.89	2.19	10.24	0.87	25.58	0.90	1.92	0.33	0.29	0.48
F5OP	1	9.20	0.57	31.73	2.14	10.60	0.94	25.43	0.87	1.92	0.39	0.30	0.46
F1OA	2	10.28	0.71	30.79	1.82	11.52	1.22	22.13	0.64	1.90	0.37	0.33	0.28
F1OP	2	10.47	0.73	30.61	1.81	11.43	1.38	21.73	0.65	1.75	0.28	0.33	0.25
F2OA	2	9.90	0.66	30.89	2.00	10.72	1.20	23.72	0.76	1.85	0.34	0.31	0.37
F2OP	2	9.77	0.65	31.02	1.99	10.72	1.31	24.00	0.78	1.95	0.48	0.31	0.35
F3OA	2	9.59	0.63	31.07	2.15	10.05	1.14	25.12	0.85	1.96	0.28	0.30	0.37
F3OP	2	9.47	0.62	31.43	2.15	10.41	1.09	25.17	0.83	1.89	0.29	0.30	0.41
F4OA	2	9.29	0.59	31.49	2.19	10.19	1.07	25.83	0.86	1.94	0.32	0.30	0.45
F4OP	2	9.22	0.59	31.56	2.18	10.34	1.07	25.84	0.88	1.87	0.27	0.30	0.44
F5OA	2	9.12	0.58	31.67	2.23	10.12	1.00	26.38	0.89	1.90	0.28	0.29	0.47
F5OP	2	9.03	0.56	31.57	2.24	10.05	1.04	26.49	0.92	1.96	0.28	0.29	0.47
