# Supplementary material for: Nutritional and physicochemical quality of formulations based on colostrum and bovine whey
Source: PLoS One. 2022 May 2;17(5):e0267409. doi: 10.1371/journal.pone.0267409 (PMC9060355; doi:10.1371/journal.pone.0267409)
Supplement: S4 Table — (PDF) [file pone.0267409.s004.pdf]

	Repetition	SCFA	MCFA	n3	n6	SFA	UFA	n3_n6	TI	AI
F1OA	1	3.77	6.61	0.30	2.16	66.30	29.07	7.20	3.49	2.60
F1OP	1	3.96	7.25	0.90	2.28	66.19	29.46	2.53	3.04	2.52
F2OA	1	3.62	5.72	0.27	2.13	64.62	30.78	7.89	3.28	2.39
F2OP	1	3.63	5.83	0.32	2.32	64.29	31.17	7.25	3.19	2.34
F3OA	1	3.41	5.11	0.29	2.37	63.12	32.27	8.17	3.08	2.25
F3OP	1	3.43	4.99	0.27	2.25	63.01	32.44	8.33	3.08	2.22
F4OA	1	3.39	4.74	0.44	2.45	61.42	33.15	5.57	2.90	2.14
F4OP	1	2.50	4.33	0.36	2.41	61.24	34.15	6.69	2.88	2.04
F5OA	1	3.30	4.42	0.33	2.40	61.74	33.45	7.27	2.93	2.12
F5OP	1	3.12	4.52	0.39	2.38	61.86	33.35	6.10	2.92	2.12
F1OA	2	3.82	6.66	0.37	2.18	65.80	29.72	5.89	3.33	2.52
F1OP	2	4.08	7.04	0.28	2.00	66.40	29.24	7.14	3.43	2.59
F2OA	2	3.87	5.86	0.34	2.22	63.96	31.54	6.53	3.10	2.32
F2OP	2	3.65	5.57	0.48	2.30	63.40	32.14	4.79	2.97	2.26
F3OA	2	3.59	5.26	0.28	2.33	62.28	33.14	8.32	2.94	2.17
F3OP	2	3.37	5.03	0.29	2.30	62.38	33.08	7.93	2.98	2.17
F4OA	2	3.28	4.64	0.32	2.39	61.55	33.88	7.47	2.88	2.09
F4OP	2	3.28	4.53	0.27	2.31	61.62	33.78	8.56	2.92	2.09
F5OA	2	3.07	4.32	0.28	2.37	60.96	34.35	8.46	2.85	2.05
F5OP	2	3.28	4.24	0.28	2.43	60.82	34.58	8.68	2.82	2.02
