# Supplementary material for: Nutritional and physicochemical quality of formulations based on colostrum and bovine whey
Source: PLoS One. 2022 May 2;17(5):e0267409. doi: 10.1371/journal.pone.0267409 (PMC9060355; doi:10.1371/journal.pone.0267409)
Supplement: S5 Table — (PDF) [file pone.0267409.s005.pdf]

	Repetition	C14:0	C14:1	C16:0	C16:1	C18:0	C18:1 t11	C18:1 c9	C18:1 c11	C18:2n6	C18:3n3	CLA c9t11	C20:4n6
Colostrum_raw	1	8.70	0.52	31.98	2.33	9.76	0.91	27.15	0.98	1.96	0.28	0.26	0.55
Colostrum_Past	1	8.73	0.52	32.07	2.35	9.66	0.88	27.23	1.00	2.07	0.31	0.26	0.56
Colostrum_raw	2	8.54	0.51	31.58	2.35	9.61	1.20	28.18	0.97	2.03	0.28	0.27	0.59
Colostrum_Past	2	8.66	0.52	31.70	2.38	9.48	1.24	27.96	0.99	2.07	0.29	0.27	0.56

	Repetition	SCFA	MCFA	n3	n6	SFA	UFA	n3_n6	TI	AI
Colostrum_raw	1	3.19	3.60	0.28	2.51	59.86	35.00	8.96	2.75	1.96
Colostrum_Past	1	3.03	3.62	0.31	2.63	59.73	35.50	8.48	2.73	1.94
Colostrum_raw	2	2.80	3.42	0.28	2.62	58.56	36.77	9.36	2.61	1.84
Colostrum_Past	2	2.90	3.56	0.29	2.63	58.92	36.61	9.07	2.62	1.86
